# Mortality and causes of death in patients with Parkinson's disease: a nationwide population-based cohort study

**DOI:** 10.3389/fneur.2023.1236296

**Published:** 2023-08-31

**Authors:** Dong-Woo Ryu, Kyungdo Han, A-Hyun Cho

**Affiliations:** ^1^Department of Neurology, College of Medicine, The Catholic University of Korea, Seoul, Republic of Korea; ^2^Department of Statistics and Actuarial Science, Soongsil University, Seoul, Republic of Korea

**Keywords:** Parkinson's disease, mortality, cause of death, population characteristics, epidemiology

## Abstract

**Objective:**

Parkinson's disease (PD) is a neurodegenerative disease involving multiple systems that can affect mortality. This study aimed to compare all-cause and cause-specific mortality between people with PD and without PD.

**Methods:**

This population-based prospective cohort study is based on Korean National Health Insurance Service data. The primary outcome was the hazard ratio (HR) of all-cause and cause-specific mortality for PD from 2010 to 2019. Cox proportional hazards regression was applied to calculate HRs under crude and three adjusted models with epidemiologic variables.

**Results:**

A total of 8,220 PD patients and 41,100 age- and sex-matched controls without PD were registered. Ten-year mortality was 47.9% in PD patients and 20.3% in non-PD controls. The mortality rate was higher among older and male participants. The leading cause of death in PD was nervous system diseases (38.73%), and 97.1% of those were extrapyramidal and movement disorders, followed by circulatory diseases (15.33%), respiratory diseases (12.56%), and neoplasms (9.7%). PD contributed to an increased risk of all-cause death with an HR of 2.96 (95% CI = 2.84–3.08). HRs of death for PD were 3.07 (95% CI = 2.74–3.45) from respiratory diseases, 1.93 (95% CI = 1.75–2.13) from circulatory diseases, 2.35 (95% CI = 2.00–2.77) from external causes, and 2.69 (95% CI = 2.10–3.43) from infectious diseases.

**Conclusion:**

These results showed that PD was related to a higher risk of mortality in all ages and sexes. The leading causes of death in PD were nervous, circulatory, respiratory, infectious diseases, and external causes.

## Introduction

Parkinson's disease (PD) is a neurodegenerative disease affecting multiple systems that can cause cognitive or autonomic impairments along with motor symptoms. As the elderly population increases, PD patients and the associated socioeconomic burden have increased (1). Various motor and non-motor symptoms in PD can decrease the quality of life and shorten life expectancy. Although multiple factors, including age, sex, and comorbidities, are associated with death, PD can also affect death. Mild cognitive impairment or a high levodopa equivalent dose in early-phase PD was associated with a higher relative risk (RR) of death, and a difference in survival rates according to the PD subtype was observed, implying that PD has an association with an increased risk of death (2, 3). Assessing the characteristics and causes of death in PD can identify high-risk groups of deaths and expand our understanding of PD.

Although there are some differences among studies, the leading causes of death in PD were reported to be neurodegenerative disease, cardiovascular disease, pneumonia, and infection (4–6). PD had a higher risk of death, with an overall mortality ratio of 1.52 in a meta-analysis (7). Although older PD patients have higher mortality and more comorbidities, PD can have a greater impact on the quality of life and survival in younger patients (8). Few studies have observed large-scale populations for long periods or conducted subgroup analyses of death in PD. Data from the Korean National Health Insurance Service (NHIS) covering nearly the entire population can provide crucial epidemiologic information. Furthermore, since PD has been classified as a rare and incurable disease for partial exemption from medical costs in Korea, NHIS billing data provide relatively accurate PD group sampling.

This study aimed to compare mortality rates between PD patients and the normal population by age and sex and to analyze the risk of all-cause and cause-specific deaths based on the NHIS dataset.

## Materials and methods

### Data source

This study was based on data from the NHIS of Korea. The NHIS is a single government-operated insurance service, to which the entire Korean population is obligated to subscribe. Medical institutions claim benefits from the NHIS based on medical expenses, and billing data are accumulated in the NHIS database. NHIS data contain personal information, medical history, and demographics based on long-term billing data from the entire population.

NHIS data broadly consist of five databases, including eligibility for an insurance claim, medical history, medical examination, medical care, and long-term care for the elderly. The medical examination database includes medical examination measurement and questionnaire data for variables including lifestyle, family, and previous medical history. Large cohort studies can be performed based on this database because the medical examination is mandatory for people over 40 every 2 years. The medical history database has disease diagnosis data based on the International Classification of Disease-Tenth Revision-Clinical Modification (ICD-10-CM) and rare incurable disease codes. In addition, data related to a death can be obtained through linkage with the Korean Statistical Information Service database.

The NHIS provides relevant data to the public for academic research and policy establishment after deliberation. We obtained data from the NHIS after approval by the official review committee and utilized these data under their regulations.

### Study design and population

We conducted a population-based prospective cohort study based on the NHIS dataset. People over 40 who underwent medical health examinations in 2009 were included. Patients with PD were defined as people previously diagnosed with PD (ICD-10-CM code G20) and registered with code V124 in the rare and incurable disease registry. Registration in the rare and incurable disease is only possible if a neurologist or neurosurgeon finds the patient meets the UK PD Society's brain bank clinical diagnostic criteria for the diagnosis of PD (9). Although a diagnosis based on billing data codes may be inaccurate, a PD diagnosis in the rare incurable disease registry can overcome this weakness. Deaths among registered participants from 2010 to 2019 were investigated, and those who died within 1 year of registration in 2009 were excluded from the study. Participants with missing data were also excluded. PD patients were matched 1:5 with non-PD controls, adjusted for age and sex.

**Figure 1:**
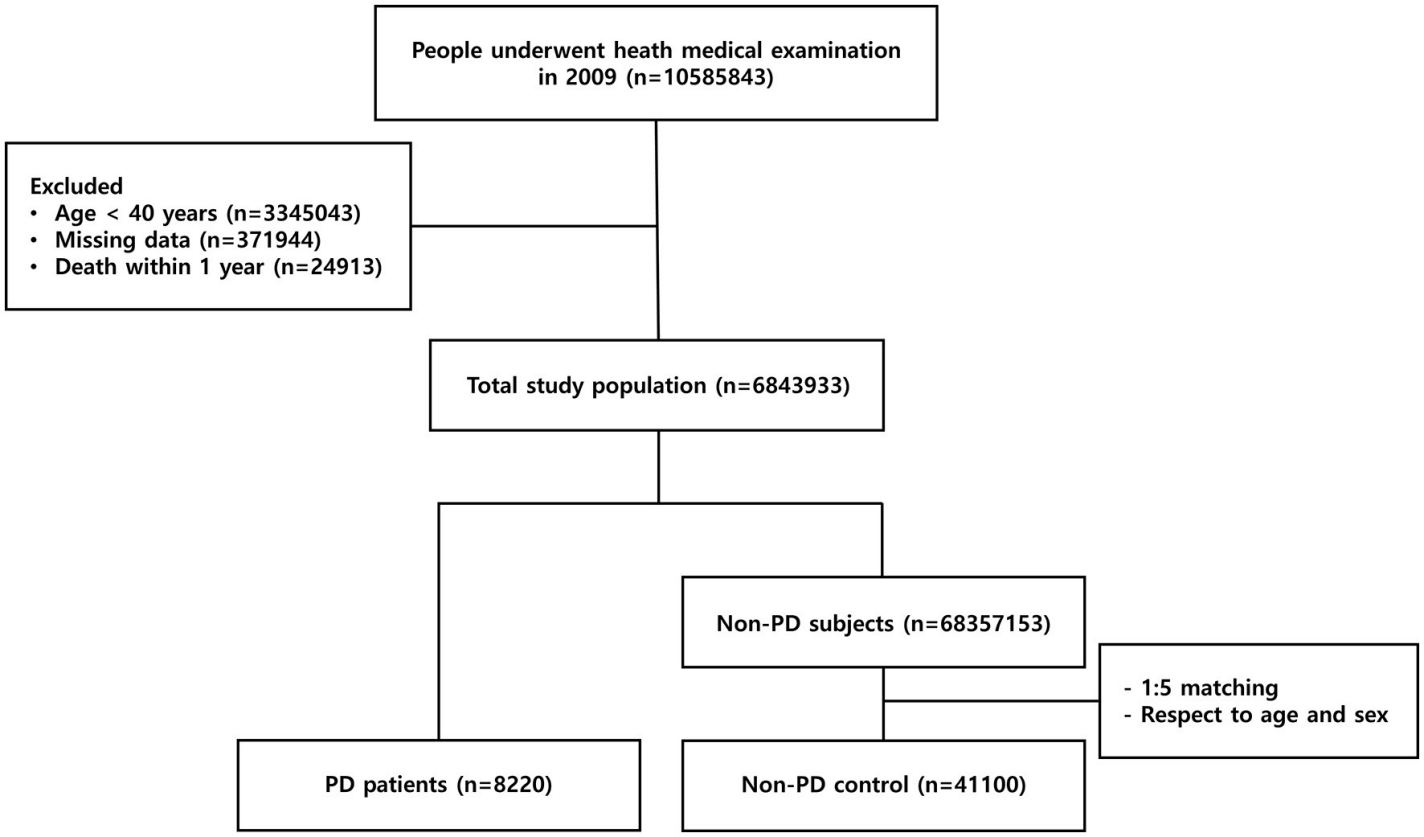
Flowchart of population enrollment in the study. PD, Parkinson's disease.

A total of 10,585,843 individuals underwent medical health examinations in 2009, of whom 6,868,856 were over 40 years old and had complete data. Since 24,943 of those died within a year, 6,843,933 individuals were included in the present study. The number of patients diagnosed with PD among the registered participants was 8,220. Among 6,835,713 non-PD subjects, 41,100 were enrolled as non-PD control (as shown in Figure 1).

The study protocol was approved by the local Institutional Ethics Committee (reference number: SC21ZISE0149), and the need for written informed consent was exempted.

### Variables

Age, sex, personal income, smoking, drinking, and physical activity data of all registered participants were collected from the NHIS dataset. We classified personal income into four quartiles and drinking into non-drinkers, mild drinkers consuming between 0 and 30 g of alcohol per day, and heavy drinkers consuming over 30 g of alcohol per day. Regular physical activity was defined as vigorous-intensity aerobic activities for at least 75 min per week, moderate-intensity aerobic activities for at least 150 min per week, or muscle-strengthening activities for two or more days per week. Comorbidities, including chronic kidney disease (CKD), DM, hypertension, hyperlipidemia, cancer, depression, ischemic heart disease (IHD), and stroke, were checked in all participants. According to the ICD-10-CM, cancer was defined as a cancer diagnosis with a C00–C96 code in the 5 years before 2009, and depression, IHD, and stroke as F32–33, I20–25, and I63–64 codes, respectively, in 2009. CKD, DM, hypertension, and hyperlipidemia were also identified based on ICM-10-CM codes, CKD: N18, N19, Z49, Z940, Z90, or Z992, DM: E11–14, hypertension: I10–13 or I15, hyperlipidemia: E78. In addition, physical measurements, blood pressure, and laboratory results were collected from the medical examination dataset.

### Outcomes

We investigated death (primary outcome), time to death, and cause of death in the registered population. Deaths from the Korean Statistical Information Service database were made based on the dying declarations of individual clinicians. It is recommended that the dying declaration report the cause of death with the highest association. According to ICD-10-CM codes, all causes of death were classified into infection (A00–B99), neoplasm (C00–D49), hematologic and immunologic diseases (D50–D89), endocrine, nutritional and metabolic diseases (E00–E88), mental disorders (F00–F99), nervous diseases (G00–G99), circulatory diseases (I00–I99), respiratory diseases (J00–J98), digestive diseases (K00–K92), musculoskeletal diseases (M00–M99), genitourinary diseases (N00–N98), unclassified (R00–R99), external causes (S00–T98), and others (H00–H59, L00–L99, O00–O9A, Q00–Q99, and Z00–Z99). Others included ophthalmic, otolaryngological, obstetric, congenital problems, and other factors influencing health status that were not related to death. Nervous system diseases as a cause of death were further analyzed for detailed causes.

### Statistical analysis

All statistical analyses were conducted with SAS software version 9.4 for Windows (SAS Institute Incorporation, Cary, NC, USA). A comparison of means was assessed using the independent *t*-test for continuous variables. The chi-square test was used to compare frequencies for categorical variables. Poisson regression models, crude and adjusted for epidemiologic factors, were used to calculate the mortality rate and 95% confidence intervals and to identify the RR of mortality in PD patients by age and sex. Cox proportional hazards regression was applied in a crude model and three adjusted models to determine the association of PD with all-cause and cause-specific mortality. The models were adjusted with the following covariates: Model 1, non-adjusted; Model 2, income, smoking, drinking, and physical activity; Model 3, income, smoking, drinking, physical activity, hypertension, DM, hyperlipidemia, and CKD; Model 4, income, smoking, drinking, physical activity, hypertension, DM, hyperlipidemia, CKD, cancer, depression, IHD, and stroke. Hazard ratios (HRs) of detailed nervous disease causes of death for PD patients were analyzed using a Cox hazard regression model in the same manner. Statistical significance was set at a *P*-value of < 0.001.

## Results

### Baseline characteristics

Clinical characteristics, comorbidities, and results of the medical health examination of PD patients and non-PD control are shown in Table 1. The mean age of PD patients was 68.2 (SD, 8.6). Male and female PD patients were 44.5 and 55.6%, respectively. PD subjects had higher incomes than non-PD control. Non-smokers and non-drinkers showed a higher proportion in PD patients than in non-PD control. People who had conducted regular physical activity were less frequent in PD patients than in non-PD control. The comorbidities including CKD (21.11%), DM (21.05%), depression (25.62%), IHD (15.45%), and stroke (21.55%) were more frequent in PD patients than in non-PD control.

**Table 1:** Clinical characteristics of PD patients and non-PD control.

**X**	**Control**	**PD patient**	***p*-value**
No. of participants	41,100	8220	
Age, mean (SD), y	68.2 (8.6)	68.2 (8.6)	
≥65 years old	28,945 (70.4)	5,789 (70.4)	
Sex
Male	18,270 (44.5)	3,654 (44.5)	
Female	22,830 (55.6)	4,566 (55.6)	
Income^*^
Level I, lowest	7,721 (18.8)
1,264 (15.4)	< 0.0001
Level 2	6,619 (16.1)	1,183 (14.4)	
Level 3	9,838 (23.9)	1,925 (23.4)	
Level 4, highest	16,922 (41.2)	3,848 (46.8)	
Smoking
Non-smoker	29,505 (71.8)	6,604 (80.3)	< 0.0001
Ex-smoker	5,988 (14.6)	1,213 (14.8)	
Current smoker	5,607 (13.6)	403 (4.9)	
Drinking^†^
Non-drinker	29,968 (72.9)	6,965 (84.7)	< 0.0001
Mild drinker	9,093 (22.1)	1,145 (13.9)	
Heavy drinker	2,039 (5.0)	110 (1.3)	
Regular physical activity^‡^	7,912 (19.3)	1,478 (18.0)	0.0074
BMI
< 18.5	1,501 (3.7)	321 (3.9)	0.0548
< 23	14,221 (34.6)	2,935 (35.7)	
< 25	10,831 (26.4)	2,079 (25.3)	
< 30	13,105 (31.9)	2,627 (32.0)	
≥30	1,442 (3.5)	258 (3.1)	
Waist circumference, mean (SD), cm	59.6 (10.2)	59.2 (10.2)	0.0005
Systolic BP, mean (SD), mmHg	128.9 (16.3)	127.2 (16.5)	< 0.0001
Diastolic BP, mean (SD), mmHg	78.0 (10.1)	77.5 (10.3)	< 0.0001
Chronic kidney disease	6,542 (15.9)	1,735 (21.1)	< 0.0001
DM	7,433 (18.1)	1,730 (21.1)	< 0.0001
Hypertension	22,325 (54.3)	4,504 (54.8)	0.4305
Dyslipidemia	12,161 (29.6)	2,473 (30.1)	0.3685
Cancer	1,377 (3.4)	296 (3.6)	0.2519
Depression	3,008 (7.3)	2,106 (25.6)	< 0.0001
Ischemic heart disease	4,707 (11.5)	1,270 (15.5)	< 0.0001
Stroke	2,410 (5.9)	1,771 (21.6)	< 0.0001
Laboratory findings
Fasting glucose, mean	102.8 (27.3)	104.9 (29.2)	< 0.0001
(SD), mg/dL			
Total cholesterol, mean (SD),	198.9 (43.9)	192.1 (54.5)	< 0.0001
mg/dL			
HDL, mean (SD), mg/dL	55.7 (37.4)	54.3 (33.4)	0.0015
LDL, mean (SD), mg/dL	119.3 (86.0)	115.5 (69.7)	0.0002
TG, median (IQR), mg/dL	122.4 (121.8–123.0)	112.8 (11.5–114.0)	< 0.0001
GFR, mean (SD),	79.9 (31.3)	76.5 (30.8)	< 0.0001
ml/min/1.73 m^2^			

PD, Parkinson disease; BMI, body mass index; BP, blood pressure; HDL, high density lipoprotein; LDL, low density lipoprotein; TG, triglyceride; GFR, glomerular filtration rate.

* Personal income was classified into four quartiles between the lowest level 1 and the highest level 4.

† Drinking habit was classified as a non-drinker, mild drinker taking between 0 and 30 grams of alcohol per day, and heavy drinker taking over 30 grams of alcohol per day.

‡ Regular physical activity was defined as vigorous-intensity aerobic activities at least for 75 min per week, moderate-intensity aerobic activities at least for 150 min per week vigorous intensity, or muscle-strengthening activities on 2 or more days per week.

On the medical examination, PD patients had shorter waist circumferences compared with non-PD control. Systolic and diastolic blood pressures were lower in PD patients than in non-PD control. In addition, all cholesterol profiles and glomerular filtration rates were lower in PD patients than in non-PD control.

### Mortality rate

**Figure 2:**
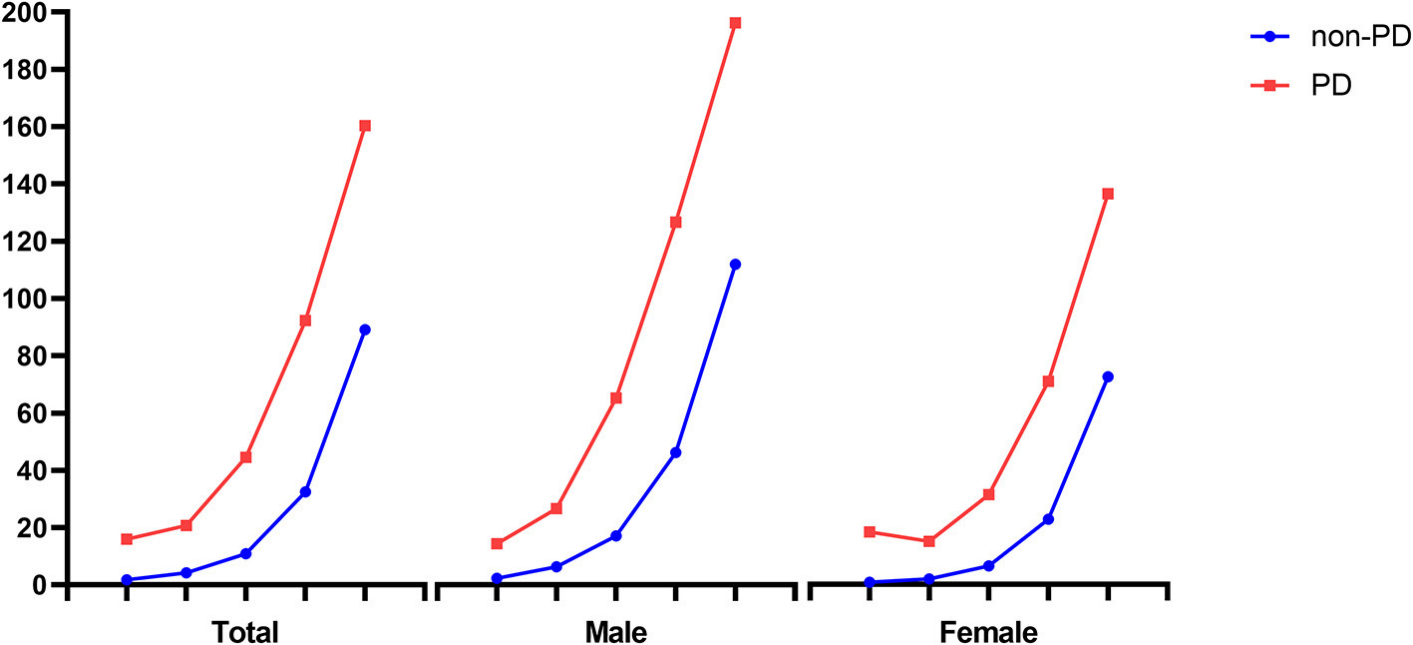
Comparison of mortality rate between PD and non-PD participants by age and sex. PD, Parkinson's disease. Mortality rate was expressed as the number of deaths per 1,000 individuals per year.

Among the 6,843,933 study population, 465,497 people died from 2010 to 2019 (6.8%). The mean duration from registration to death was 9.11 (SD, 1.28) years. The mortality rates of PD patients and non-PD control are displayed by age and sex in Table 2. PD patients showed a higher mortality rate than non-PD control of all ages and sexes. The older population had a higher mortality rate, and male participants showed a higher mortality rate at most ages (as shown in Figure 2).

**Table 2:** Comparison of mortality rate between PD patients and non-PD control by age and sex.

	**Age groups**	**Total number**		**Death**		**Crude mortality rate (95% CI)**		**Relative risk (95% CI)**	**Adjusted mortality rate (95% CI)**		**Relative risk (95% CI)**
		**Non-PD**	**PD**	**Non-PD**	**PD**	**Non-PD**	**PD**		**Non-PD**	**PD**	
Total	40–49	1,140	228	18	32	1.7 (1.07–2.7)	15.91 (11.25–22.5)	9.37 (5.26–16.69)	1.57 (0.97–2.53)	10.37 (7.33–14.68)	8.86 (4.73–16.59)
	50–59	5,225	1,045	199	187	4.13 (3.59–4.74)	20.67 (17.91–23.86)	5.01 (4.1–6.12)	3.44 (2.95–4.01)	14.68 (12.72–16.95)	4.67 (3.73–5.85)
	60–69	13,650	2,730	1,346	981	10.86 (10.3–11.46)	44.57 (41.87–47.45)	4.1 (3.78–4.45)	9.18 (8.65–9.74)	31.5 (29.57–33.55)	4.1 (3.74–4.49)
	70–79	17,995	3,599	4,904	2,228	32.41 (31.52–33.33)	92.25 (88.5–96.16)	2.85 (2.71–2.99)	29.99 (29.1–30.89)	71.46 (68.52–74.52)	2.7 (2.55–2.85)
	≥80	3,090	618	1,859	512	89.14 (85.18–93.28)	160.3 (147–174.9)	1.8 (1.63–1.98)	89.27 (85.23–93.49)	149.6 (137.1–163.2)	1.68 (1.51–1.86)
Male	40–49	710	142	15	18	2.28 (1.37–3.78)	14.39 (9.06–22.83)	6.32 (3.19–12.54)	1.9 (0–1.16E+302)	11.47 (7.22–18.21)	5.97 (2.79–12.74)
	50–59	2,600	520	150	117	6.31 (5.38–7.41)	26.63 (22.22–31.92)	4.22 (3.31–5.37)	5.74 (4.84–6.81)	22.67 (18.91–27.19)	4.2 (3.18–5.53)
	60–69	5,775	1,155	871	560	17.08 (15.98–18.25)	65.23 (60.04–70.86)	3.82 (3.44–4.25)	15.93 (14.85–17.09)	50.93 (46.86–55.36)	3.82 (3.39–4.31)
	70–79	7,780	1,556	2,861	1,168	46.25 (44.59–47.98)	126.7 (119.6–134.2)	2.74 (2.56–2.93)	45.12 (43.45–46.86)	104.2 (98.36–110.4)	2.57 (2.38–2.76)
	≥80	1,405	281	980	250	112 (105.2–119.3)	196.3 (173.4–222.2)	1.75 (1.53–2.01)	113.9 (106.9–121.3)	184.6 (163–209.1)	1.59 (1.37–1.84)
Female	40–49	430	86	3	14	0.75 (0.24–2.32)	18.42 (10.91–31.1)	24.62 (7.08–85.67)	–	11.54 (6.82–19.51)	21.18 (5.46–82.09)
	50–59	2,625	525	49	70	2 (1.51–2.65)	15.05 (11.9–19.02)	7.51 (5.22–10.83)	1.83 (1.37–2.44)	10.37 (8.2–13.13)	5.74 (3.87–8.5)
	60–69	7,875	1,575	475	421	6.52 (5.96–7.13)	31.36 (28.5–34.5)	4.81 (4.22–5.49)	5.93 (5.39–6.52)	19.37 (17.59–21.34)	4.5 (3.9–5.18)
	70–79	10,215	2,043	2,043	1,060	22.84 (21.87–23.85)	70.99 (66.84–75.39)	3.11 (2.89–3.35)	21.65 (20.69–22.65)	51.66 (48.61–54.91)	2.86 (2.64–3.09)
	≥80	1,685	337	879	262	72.59 (67.95–77.56)	136.5 (120.9–154.1)	1.88 (1.64–2.16)	72.18 (67.49–77.21)	129.9 (115.1–146.7)	1.8 (1.55–2.08)

PD, Parkinson's disease.

Mortality rate was defined as the number of deaths per 1,000 individuals per year.

Mortality rate and relative risk were calculated using Poisson regression models, both crude and adjusted for income, smoking, drinking, physical activity, hypertension, DM, hyperlipidemia, chronic kidney disease, cancer, depression, ischemic heart disease, and stroke.

In this study, 10-year mortality in PD patients and non-PD control was 47.9 and 20.3%, respectively. In particular, the mortality rate of PD patients over 80 was the highest, 160.3 (95% CI: 147–174.9) in the crude model, and 149.6 (95% CI: 137.1–163.2) in the adjusted model. Excluding the 40s, male patients with PD showed a higher mortality rate than female patients with PD in both crude and adjusted models. Although younger PD patients had a lower mortality rate than older PD patients, the RR of death was higher in younger PD patients. Among all age groups with PD, the 40s age group showed the lowest mortality rate with 15.91 (95% CI: 11.23–22.5) in the crude model and 10.37 (95% CI: 7.33–14.68) in the adjusted model, and the highest RR of death with 9.37 (95% CI: 5.26–16.69) in the crude model and 8.86 (95% CI: 4.73–16.59) in the adjusted model.

### Causes of death

All causes of mortality and their proportion of total deaths in PD patients and non-PD control are displayed in Table 3. In the non-PD population, the leading cause of death was neoplasm (32.38%), followed by circulatory diseases (21.6%), respiratory diseases (12.3%), and external causes (12.3%). In PD patients, the leading cause of death was nervous system diseases (38.73%), followed by circulatory diseases (15.33%), respiratory diseases (12.56%), and neoplasms (9.7%). Most nervous system diseases for PD deaths were extrapyramidal and movement disorders (G20–G26) (97.3%), including PD. Detailed nervous system disease causes and their mortality rates are shown in the Supplementary Table.

**Table 3:** Causes of death in PD patients and non-PD control.

	**Number**			**Percent, %**		
	**Total**	**Non-PD**	**PD**	**Total**	**Non-PD**	**PD**
Surviving	37,054	32,774	4,280	75.13	79.74	52.07
All-cause death	12,266	8,326	3,940	24.87	20.26	47.93
	49,320	41,100	8,220	100	100	100
Certain infectious and parasitic diseases (A00–B99)	349	241	108	2.85	2.89	2.74
Neoplasm (C00–D48)	3,078	2,696	382	25.09	32.38	9.7
Diseases of the blood and blood-forming organs and certain disorders involving the immune mechanism (D50–D89)	30	25	5	0.24	0.3	0.13
Endocrine, nutritional, and metabolic diseases (E00–E88)	388	282	106	3.16	3.39	2.69
Mental, behavioral, and neurodevelopmental disorders (F00–F99)	138	118	20	1.13	1.42	0.51
Diseases of the nervous system (G00–G99)	1,828	302	1,526	14.9	3.63	38.73
Diseases of the circulatory system (I00–I99)	2,402	1,798	604	19.58	21.6	15.33
Diseases of the respiratory system (J00–J98)	1,519	1,024	495	12.38	12.3	12.56
Diseases of the digestive system (K00–K92)	351	271	80	2.86	3.25	2.03
Diseases of the musculoskeletal system and connective tissue (M00–M99)	65	48	17	0.53	0.58	0.43
Diseases of the genitourinary system (N00–N98)	257	189	68	2.1	2.27	1.73
Symptoms, signs, and abnormal clinical and laboratory findings, not elsewhere classified (R00–R99)	947	673	274	7.72	8.08	6.95
Injury, poisoning, and certain other consequences of external causes (S00–T98)	842	610	232	6.86	7.33	5.89
Other (H00–H59, H60–H95, L00–L99, O00–O9A, Q00–Q99, Z00–U99)^*^	27	19	8	0.22	0.23	0.2
Missing death	45	30	15	0.37	0.36	0.38
	12,266	8,326	3,940	100	100	100

PD, Parkinson's disease.

^*^Other included ophthalmic, otolaryngological, obstetric, congenital problems, and factors influencing health status, which were less frequent causes of death.

### Hazard ratio of death

**Figure 3:**
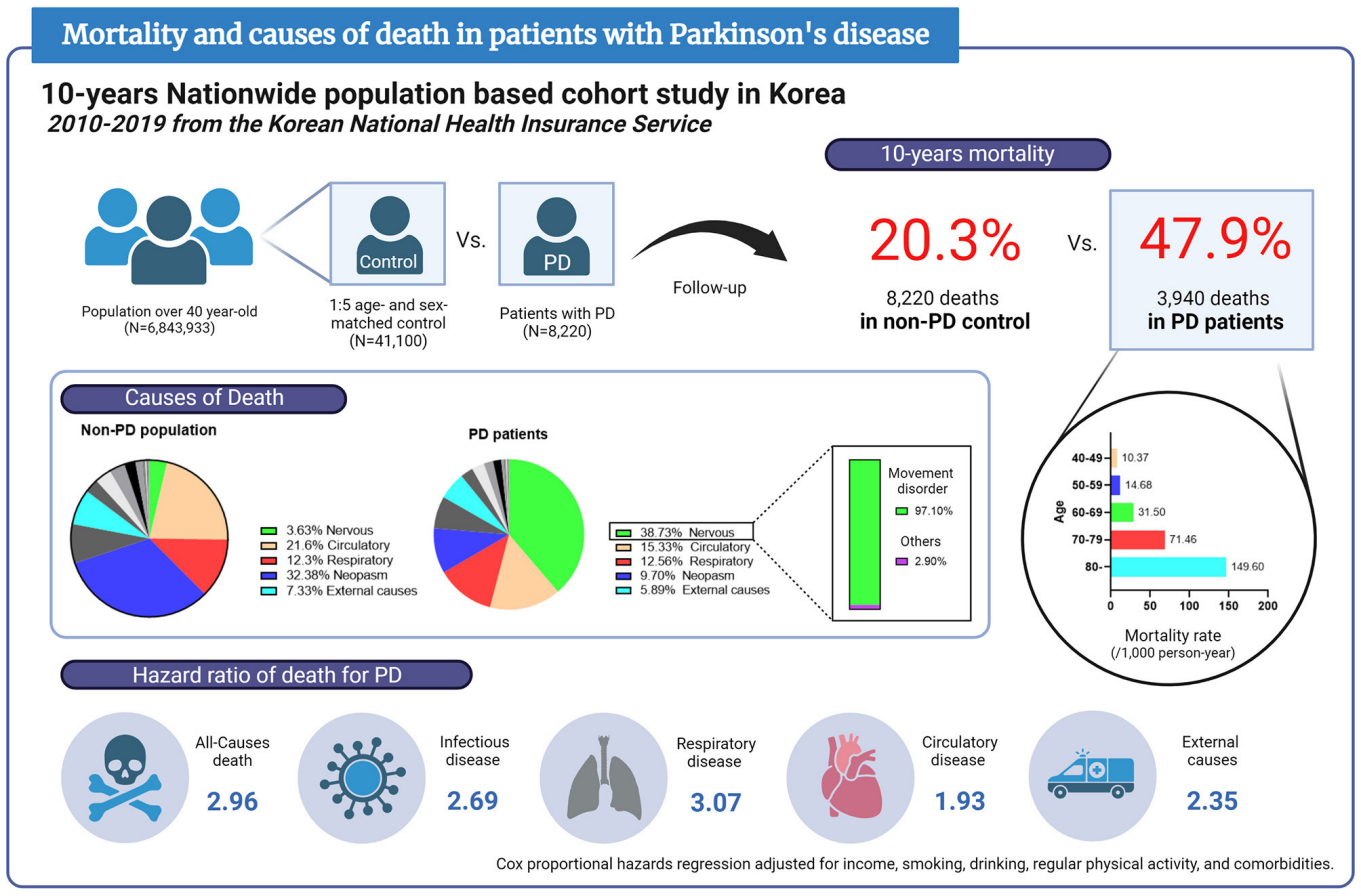
Infographic of mortality and causes of death in patients with Parkinson's disease based on this report. PD, Parkinson's disease.

Mortality rate and HR of all-cause and cause-specific death for PD are shown in Table 4 and Figure 3. The all-cause mortality rates in PD patients and non-PD control were 65.21 and 23.50 deaths per 1,000 persons per year, respectively. PD had an association with increased risk of all-cause death in the crude and adjusted models [HR (95% CI), 2.88 (2.77–2.99) in model 1; 3.24 (3.11–3.37) in model 2; 3.17 (3.05–3.30) in model 3; 2.96 (2.84–3.08) in model 4]. In addition, PD was correlated with an increased risk of death from all detailed causes, except hematologic and immune-related and mental disorders, in the crude model. In adjusted models, the risks of death from the infectious, endocrine and metabolic, nervous system, circulatory, respiratory, digestive, musculoskeletal, genitourinary, unclassified, external, and other causes were increased in PD patients. However, PD was not associated with an increased risk of death from neoplasms, hematologic and immune-related or mental disorders. Excluding death from nervous system diseases, the HR of death from respiratory diseases was highest in the adjusted model in PD patients [HR (95% CI), 3.07 (2.74–3.45)]. Among other major causes of death in the adjusted model, the HRs (95% CI) of death for PD were 1.93 (1.75–2.13) from circulatory diseases, 2.35 (2.00–2.77) from external causes, and 2.58 (2.13–3.13) for death from infectious diseases.

**Table 4:** Mortality rates and hazard ratios for all-cause and cause-specific deaths in PD.

	**PD**	**Number**	**Death**	**Duration, person-years**	**Mortality rate^‡^**	**Model 1**		**Model 2**		**Model 3**		**Model 4**	
						**Hazard ratio (95% CI)**	***P*** **-value**	**Hazard ratio (95% CI)**	***P*** **-value**	**Hazard ratio (95% CI)**	***P*** **-value**	**Hazard ratio (95% CI)**	***P*** **-value**
All causes of death	No	41,100	8,326	354,903.85	23.4599	1 (ref.)	< 0.0001	1 (ref.)	< 0.0001	1 (ref.)	< 0.0001	1 (ref.)	< 0.0001
	Yes	8,220	3,940	60,413.61	65.2171	2.877 (2.770, 2.988)		3.237 (3.114, 3.366)		3.173 (3.052, 3.300)		2.960 (2.840, 3.084)	
Infectious (A00–B99)	No	41,100	241	354,903.85	0.67906	1 (ref.)	< 0.0001	1 (ref.)	< 0.0001	1 (ref.)	< 0.0001	1 (ref.)	< 0.0001
	Yes	8,220	108	60,413.61	1.78768	2.766 (2.204, 3.472)		3.087 (2.448, 3.893)		2.971 (2.354, 3.750)		2.685 (2.100, 3.433)	
Neoplasm (C00–D48)	No	41,100	2,696	354,903.85	7.59642	1 (ref.)	0.0021	1 (ref.)	0.66	1 (ref.)	0.5962	1 (ref.)	0.3073
	Yes	8,220	382	60,413.61	6.32308	0.845 (0.759, 0.941)		0.976 (0.875, 1.088)		0.971 (0.871, 1.083)		0.943 (0.842, 1.056)	
Hematologic and immune (D50–D89)	No	41,100	25	354,903.85	0.070442	1 (ref.)	0.7102	1 (ref.)	0.5652	1 (ref.)	0.6927	1 (ref.)	0.6555
	Yes	8,220	5	60,413.61	0.082763	1.200 (0.459, 3.137)		1.330 (0.503, 3.514)		1.217 (0.459, 3.225)		1.258 (0.460, 3.442)	
Endocrine and metabolic (E00–E88)	No	41,100	282	354,903.85	0.79458	1 (ref.)	< 0.0001	1 (ref.)	< 0.0001	1 (ref.)	< 0.0001	1 (ref.)	< 0.0001
	Yes	8,220	106	60,413.61	1.75457	2.249 (1.798, 2.813)		2.488 (1.981, 3.126)		2.133 (1.699, 2.678)		2.001 (1.576, 2.541)	
Mental (F00–F99)	No	41,100	118	354,903.85	0.33248	1 (ref.)	0.8626	1 (ref.)	0.5025	1 (ref.)	0.6845	1 (ref.)	0.7126
	Yes	8,220	20	60,413.61	0.33105	1.043 (0.649, 1.676)		1.178 (0.730, 1.902)		1.105 (0.683, 1.787)		0.911 (0.554, 1.498)	
Nervous (G00–G99)	No	41,100	302	354,903.85	0.8509	1 (ref.)	< 0.0001	1 (ref.)	< 0.0001	1 (ref.)	< 0.0001	1 (ref.)	< 0.0001
	Yes	8,220	1,526	60,413.61	25.2592	31.053 (27.443, 35.138)		32.015 (28.233, 36.303)		32.262 (28.444, 36.593)		30.395 (26.711, 34.588)	
Circulatory (I00–I99)	No	41,100	1,798	354,903.85	5.06616	1 (ref.)	< 0.0001	1 (ref.)	< 0.0001	1 (ref.)	< 0.0001	1 (ref.)	< 0.0001
	Yes	8,220	604	60,413.61	9.99775	2.039 (1.859, 2.236)		2.259 (2.057, 2.482)		2.181 (1.985, 2.396)		1.932 (1.750, 2.133)	
Respiratory (J00–J98)	No	41,100	1,024	354,903.85	2.88529	1 (ref.)	< 0.0001	1 (ref.)	< 0.0001	1 (ref.)	< 0.0001	1 (ref.)	< 0.0001
						**Hazard ratio (95% CI)**	***P*** **-value**	**Hazard ratio (95% CI)**	***P*** **-value**	**Hazard ratio (95% CI)**	***P*** **-value**	**Hazard ratio (95% CI)**	***P*** **-value**
	Yes	8,220	495	60,413.61	8.19352	3.025 (2.717, 3.368)		3.445 (3.086, 3.846)		3.385 (3.031, 3.780)		3.073 (2.735, 3.452)	
Digestive (K00–K92)	No	41,100	271	354,903.85	0.76359	1 (ref.)	< 0.0001	1 (ref.)	< 0.0001	1 (ref.)	< 0.0001	1 (ref.)	< 0.0001
	Yes	8,220	80	60,413.61	1.3242	1.789 (1.393, 2.296)		2.071 (1.606, 2.672)		2.009 (1.557, 2.594)		1.811 (1.384, 2.370)	
Musculoskeletal (M00–M99)	No	41,100	48	354,903.85	0.13525	1 (ref.)	0.0081	1 (ref.)	0.0047	1 (ref.)	0.0049	1 (ref.)	0.0121
	Yes	8,220	17	60,413.61	0.28139	2.114 (1.215, 3.678)		2.244 (1.282, 3.927)		2.241 (1.277, 3.930)		2.130 (1.180, 3.845)	
Genitourinary (N00–N98)	No	41,100	189	354,903.85	0.53254	1 (ref.)	< 0.0001	1 (ref.)	< 0.0001	1 (ref.)	< 0.0001	1 (ref.)	< 0.0001
	Yes	8,220	68	60,413.61	1.12557	2.227 (1.687, 2.939)		2.545 (1.918, 3.375)		2.250 (1.695, 2.985)		2.029 (1.508, 2.731)	
Unclassified (R00–R99)	No	41,100	673	354,903.85	1.89629	1 (ref.)	< 0.0001	1 (ref.)	< 0.0001	1 (ref.)	< 0.0001	1 (ref.)	< 0.0001
	Yes	8,220	274	60,413.61	4.5354	2.530 (2.198, 2.912)		2.922 (2.532, 3.373)		2.898 (2.510, 3.346)		2.864 (2.463, 3.331)	
External (S00–T98)	No	41,100	610	354,903.85	1.71878	1 (ref.)	< 0.0001	1 (ref.)	< 0.0001	1 (ref.)	< 0.0001	1 (ref.)	< 0.0001
	Yes	8,220	232	60,413.61	3.84019	2.249 (1.933, 2.617)		2.514 (2.153, 2.937)		2.506 (2.145, 2.929)		2.354 (1.997, 2.774)	
Other^†^	No	41,100	19	354,903.85	0.05354	1 (ref.)	0.0258	1 (ref.)	0.0299	1 (ref.)	0.0344	1 (ref.)	0.0297
	Yes	8,220	8	60,413.61	0.13242	2.562 (1.121, 5.858)		2.525 (1.095, 5.827)		2.471 (1.069, 5.715)		2.645 (1.101, 6.357)	

PD, Parkinson's disease.

Causes of death was defined using International Classification of Disease-Tenth Revision-Clinical modification codes (A00–B99, certain infectious and parasitic disease; C00–D48, neoplasm; D50–D89, diseases of the blood and blood-forming organs and certain disorders involving the immune mechanism; E00–E88, endocrine, nutritional, and metabolic diseases; F00–F99, mental, behavioral and neurodevelopmental disorders; G00–G99, diseases of the nervous system; I00–I99, diseases of the circulatory system; J00–J98, diseases of the respiratory system; K00–K92, diseases of the digestive system; M00–M99, diseases of the musculoskeletal system and connective tissue; N00–N98, diseases of the genitourinary system; R00–R99, symptoms, signs, and abnormal clinical and laboratory findings, not elsewhere classified; S00–T98, injury, poisoning, and certain other consequences of external causes).

^†^Other included ophthalmic, otolaryngological, obstetric, and congenital problems.

^‡^Mortality rate was defined as the number of deaths per 1,000 individuals per year. Cox proportional hazards regression was performed to calculate hazard ratios with four models (Model 1, crude; Model 2, adjusted for income, smoking, drinking, and regular physical activity; Model 3, adjusted for income, smoking, drinking, regular physical activity, DM, hypertension, hyperlipidemia, and chronic kidney disease; Model 4, adjusted for income, smoking, drinking, regular physical activity, DM, hypertension, hyperlipidemia, chronic kidney disease, cancer, depression, ischemic heart disease, and stroke).

## Discussion

This large-scale study compared PD patients and non-PD control based on the NHIS data from 6,843,933 population representing the South Korean population over 40 years old. The present study investigated income, physical activity, smoking, drinking, comorbidities, and various physical measurements among the registered population. This study demonstrated that PD was associated with an increased risk of death, and nervous system diseases, respiratory diseases, circulatory diseases, and external causes were the leading causes of death in PD patients.

The overall prevalence of PD in the total population is 0.12% in this study. In a meta-analysis, the age-standardized prevalence of PD was approximately 0.4–0.8% in 2010 in the US population over 45 years of age (10). PD prevalence varied from country to country and was highest in high-income North Americans (1). Although PD prevalence in this study is lower than in North Americans, those of 0.55% at 70 and 0.62% at 80 were similar. The number of PD patients is increasing worldwide and doubled in 2015 compared to 1990, reaching over 6 million worldwide (1, 11). Since good epidemiological studies showing this increase are insufficient, the prevalence information in this study based on data representing the Korean population is valuable.

In this study, female participants were more common than male participants in PD patients. A previous meta-analysis revealed that PD incidence was higher in male participants compared with female participants (12). They reported that the PD incidence rate (per 100,000 person-years) between the ages of 60 and 69 was 58.22 in male participants and 30.32 in female participants, while it was 162.58 in male participants and 93.32 in female participants between the ages of 70 and 79. In our study, younger PD patients with lower mortality had more male participants than female participants, and older PD patients with higher mortality had more female participants than male participants. Therefore, the higher proportion of female participants in PD patients may be caused by the higher mortality of male participants. PD prevalence became similar between male participants and female participants at an advanced age (13), implying that the aging phenomenon in Korea can affect our results. Differences in environmental and hormonal factors can cause sex differences in PD (14). In addition, genetic, ethnic, and social factors among Koreans could influence PD prevalence according to sex.

The proportion of non-smokers was higher in PD patients than in non-PD subjects in the present study, corresponding with the previously reported negative association of smoking with PD. A meta-analysis showed a 2-fold risk reduction of PD in cigarette smokers compared with non-smokers (15). In this study, non-drinkers were more frequent in PD patients than in non-PD subjects. Although some reports demonstrated that alcohol consumption was associated with a reduction in PD risk (16), the association between drinking and PD is still controversial.

The prevalence of diabetes and mean serum fasting glucose were significantly higher in PD patients than in non-PD participants. Although BMI was not significantly different between PD and non-PD groups, waist circumference in the medical examination was shorter in PD patients. Diabetes and prediabetes increased the risk of PD by 27 and 4%, respectively, as compared to those without diabetes (17). Type 2 diabetes can enhance neurodegeneration through reduced brain insulin signaling, which can be more facilitated in patients with a lower BMI (18). Furthermore, an observational study showed that PD patients with diabetes were more severe in motor and non-motor symptoms and had a faster progression (19).

The total cholesterol profile was lower among PD patients, which the use of lipid-lowering agents or changes in eating habits after PD development may cause. The risk of PD decreased with increasing self-reported cholesterol but was not associated with a history of hypercholesterolemia in the Nurses' Health Study and Health Professionals' Follow-up Study cohorts (20). Although the use of lipid-lowering agents was not analyzed in this study, a contrasting association between statins and the risk of PD development has been reported (21, 22). Further investigation is needed on the correlation between cholesterol levels, the use of lipid-lowering agents, and PD development. Although the prevalence of hypertension and dyslipidemia was not different between PD and non-PD groups, the history of CKD, IHD, and stroke was more frequent among PD patients. Therefore, it is necessary to consider whether these comorbidities can affect PD development or, in contrast, whether the prodromal PD state may be associated with these comorbidities.

In this study, the RR of death for PD increased in all ages. Mortality was higher in older age and male sex, so the mortality rate in male participants with PD over 80 years of age reached 184.6 per 1,000 person-years. However, the RR of death for PD was higher in younger or female patients, which may imply that age or sex could not make much difference in the effect of PD on death. Due to the difference in mortality according to age and sex in the total population, the impact of PD on deaths may have seemed larger in younger or female participants. In addition, because younger PD patients may have a longer disease duration, the impact of PD on mortality and quality of life can be more extensive (8).

This study's most common cause of death in the total population was cancer, but it was a nervous system disease in PD patients. Although most causes of death increased among PD patients, cancer did not significantly increase compared to non-PD participants. A meta-analysis reported that PD had a negative association with lung, genitourinary, colon, and hematological cancers and a positive association with melanoma and brain cancer (23). It may depend on individual cancers, but deaths from total cancers did not significantly increase in PD patients. Dissimilar to previous studies, a cohort study using Taiwan national health insurance data found more prevalent cancer in PD, which may have been influenced by the healthcare system as well as ethnicity, environmental, and genetic factors, so attention should be paid to interpretation (24).

In contrast, death from diseases of the nervous system increased with an HR of 31.169 in PD patients, with extrapyramidal and movement disorders accounting for 97.3% of those diseases. Other nervous systemic causes of death, except for extrapyramidal and movement disorders, were rare. PD has been reported as the cause of death in 18.2% of total PD deaths in the previous population-based study (25, 26). However, PD deaths from extrapyramidal and movement disorders accounted for 37.6% of total PD deaths in this study, which was higher than in previous studies. It may be because the cause of death was determined at various facilities in our research, including large populations from the claim data. An evaluation for determining a cause of death and its criteria may have varied in each case, which can be a limitation of this study.

The HR of all-cause death in PD patients showed an ~3-fold increase in the adjusted model with age, clinical characteristics, and comorbidities. Among the causes of death, respiratory diseases increased the most in PD patients, except for nervous system diseases. PD can increase the risk of aspiration due to swallowing disturbances. Furthermore, low physical activity, decreased respiratory function, and facility admissions can heighten the risk of acute respiratory syndrome in PD patients (27, 28). In a population-based study, pneumonia was the cause of death most frequently associated with PD (29). The HR of death for respiratory diseases was higher in Lewy body dementia than in Alzheimer's disease or vascular dementia (30).

Furthermore, the HR of death from certain infectious and parasitic diseases increased in PD patients. Systemic infections can affect PD deterioration through altered drug pharmacodynamics, changes in dopaminergic metabolism or signaling, or their neuronal toxic effects (31). An observational study reported that although the incidence of infection in the respiratory and urinary tract increased in PD, the risk of death in septic patients with PD was lower than in those without PD (32). Therefore, the increase in mortality from infectious diseases in PD patients may have been purely due to the increase in infection incidence.

Mortality from diseases of the circulatory system increased in PD patients. Circulatory diseases, next to those from nervous system diseases, were the second highest cause of death in PD patients. In numerous population-based epidemiological studies, circulatory diseases were the leading cause of death in PD (4, 6, 30). Dysautonomia can affect cardiovascular functions in PD patients, increasing mortality from circulatory diseases (33). PD shares many risk factors with cardiovascular disease in genetic, metabolic, and cellular pathways (34). In addition, PD hindering physical activities can decrease cardiovascular function (35). In contrast to death from respiratory diseases prevalent in advanced-stage PD, death from circulatory diseases was highly prevalent even in mild to moderate stages (36). Therefore, since PD diagnosis, detailed monitoring of cardiovascular functions is required to reduce mortality.

External causes, one of the leading causes of death in PD, refer to environmental events or circumstances that can cause injury, including transport accidents, trauma, intended self-harm, and assault. Postural instability and gait disturbance in PD increase the risk of falls and bone fractures (37, 38). Driving capability can be impaired due to cognitive and motor symptoms, increasing the risk of car accidents, especially in late-stage PD (39). Exercise, rehabilitation, medical treatment of motor and non-motor symptoms, and avoiding high-risk activities are needed to prevent these secondary injuries.

The limitations of the present study are as follows. First, as described above, the billing data for benefits claims may include inaccurate data related to diagnosis. Therefore, we eliminated inaccurate or incomplete variables and classified diagnoses of specific diseases based on widely used operational definitions. Second, this study could not determine the detailed mechanism of death from extrapyramidal and movement disorders. Death caused by extrapyramidal and movement disorders may be heterogeneous and be accompanied by other causes of death. Further investigations related to deaths from PD are needed. Third, this study did not evaluate disease durations, disease severity, or medical treatments for death in PD. Those factors can affect mortality and may be related to the cause of death. An epidemiological study reported that levodopa treatment could delay death in PD (40). Since billing data have limitations for assessing clinical outcomes, further investigations with clinical data are needed to clarify the associations between clinical characteristics and death in PD. Fourth, there were differences in clinical characteristics between PD and non-PD control. In the statistical analyses, four models adjusted with clinical variables were built to compare the groups. Finally, the present study was based on NHIS data from Koreans, so most of the study population was Asian. The results of our study may not be generalizable to other ethnicities.

## Conclusion

This population-based study based on the Korean NHIS data provided PD prevalence and its characteristics in the Korean population. We conclude that PD could increase mortality, and the leading causes of death in PD were nervous, circulatory, respiratory, infectious diseases, and external causes. Research on mortality in patients with PD could help understand the disease's course and reduce mortality. Further study and systematic management of PD patients are required to prevent death in PD.

## Data availability statement

The data analyzed in this study was obtained from the Korean National Health Insurance Service (NHIS), the following licenses/restrictions apply: these datasets must only be used for authorized purposes. Requests to access these datasets should be directed to: KH, hkd917@naver.com.

## Author contributions

D-WR, KH, and A-HC contributed to the conception and design of the manuscript. D-WR and KH contributed to the acquisition of clinical data and contributed to the analysis of data. D-WR drafted the manuscript. KH and A-HC revised the manuscript. All authors read and approved the final version of the manuscript for publication.
