# Supplementary material for: Mortality and causes of death in patients with Parkinson's disease: a nationwide population-based cohort study
Source: Front Neurol. 2023 Aug 31;14:1236296. doi: 10.3389/fneur.2023.1236296 (PMC10501780; doi:10.3389/fneur.2023.1236296)
Supplement: Supplementary file 1 [file Table_1.DOCX]

Supplementary Material

Mortality and causes of death in patients with Parkinson’s disease: A nationwide population-based cohort study

Dong-Woo Ryu, Kyungdo Han*, A-Hyun Cho*

*** Correspondence:** A-Hyun Cho: [ahyun@catholic.ac.kr](mailto:ahyun@catholic.ac.kr), Kyungdo Han: hkd917@naver.c

# Supplementary tables

Supplementary Table. Mortality rates and hazard ratios of death from a specific nervous disease.

Abbreviations: PD, Parkinson disease

*Causes of deaths were expressed as simple words with the code of International Classification of Disease-Tenth Revision-Clinical modification (G00-G09, Inflammatory diseases of the central nervous system; G10-G14, Systemic atrophies primarily affecting the central nervous system; G20-G26, Extrapyramidal and movement disorders; G30-G32, Other degenerative diseases of the nervous system; G35-G37, Demyelinating diseases of the central nervous system; G40-G47, Episodic and paroxysmal disorders; G50-G59, Nerve, nerve root and plexus disorders; G60-G64, Polyneuropathies and other disorders of the peripheral nervous system; G70-G73, Diseases of myoneural junction and muscle; G80-G83, Cerebral palsy and other paralytic syndromes; G90-G99: Other disorders of the nervous system).

†Mortality rate was defined as number of deaths per 1000 individuals per year. Cox proportional hazards regression was performed for calculate hazard ratio under 4 models (Model 1, crude; Model 2, adjusted for income, smoking, drinking, and regular physical activity; Model 3, adjusted for income, smoking, drinking, regular physical activity, DM, hypertension, hyperlipidemia and chronic kidney disease; Model 4, adjusted for income, smoking, drinking, regular physical activity, DM, hypertension, hyperlipidemia, chronic kidney disease, cancer, depression, ischemic heart disease, and stroke

|  | **PD** | **Number** | **Death** | **Duration, person-years** | **Mortality Rate**^†^ | **Model 1** | | **Model 2** | | **Model 3** | | **Model 4** | |
| --- | --- | --- | --- | --- | --- | --- | --- | --- | --- | --- | --- | --- | --- |
|  |  |  |  |  |  | **Hazard Ratio (95% C.I)** | ***P*-value** | **Hazard Ratio (95% C.I)** | ***P*-value** | **Hazard Ratio (95% C.I)** | ***P*-value** | **Hazard Ratio (95% C.I)** | ***P*-value** |
| Inflammatory (G00-09) | No | 41100 | 6 | 354903.85 | 0.016906 | 1 (ref.) | - | 1 (ref.) | - | 1 (ref.) | - | 1 (ref.) | - |
|  | Yes | 8220 | 0 | 60413.61 | 0 | - |  | - |  | - |  | - |  |
| Atrophic (G10-14) | No | 41100 | 7 | 354903.85 | 0.019724 | 1 (ref.) | 0.0083 | 1 (ref.) | 0.0129 | 1 (ref.) | 0.0104 | 1 (ref.) | 0.0181 |
|  | Yes | 8220 | 5 | 60413.61 | 0.082763 | 4.702 (1.489, 14.848) |  | 4.473 (1.373, 14.572) |  | 4.700 (1.439, 15.357) |  | 4.548 (1.296, 15.964) |  |
| Movement (G20-26) | No | 41100 | 103 | 354903.85 | 0.2902 | 1 (ref.) | <.0001 | 1 (ref.) | <.0001 | 1 (ref.) | <.0001 | 1 (ref.) | <.0001 |
|  | Yes | 8220 | 1482 | 60413.61 | 24.5309 | 87.772 (71.880, 107.177) |  | 87.949 (71.912, 107.563) |  | 88.730 (72.536, 108.539) |  | 83.095 (67.772, 101.883) |  |
| Degenerative (G30-32) | No | 41100 | 163 | 354903.85 | 0.45928 | 1 (ref.) | 0.4914 | 1 (ref.) | 0.1346 | 1 (ref.) | 0.1392 | 1 (ref.) | 0.104 |
|  | Yes | 8220 | 29 | 60413.61 | 0.48002 | 1.149 (0.774, 1.706) |  | 1.356 (0.910, 2.021) |  | 1.353 (0.906, 2.018) |  | 1.409 (0.932, 2.129) |  |
| Demyelinating (G35-37) | No | 41100 | 2 | 354903.85 | 0.005635 | 1 (ref.) | - | 1 (ref.) | - | 1 (ref.) | - | 1 (ref.) | - |
|  | Yes | 8220 | 0 | 60413.61 | 0 | - |  | - |  | - |  | - |  |
| Episodic (G40-47) | No | 41100 | 9 | 354903.85 | 0.025359 | 1 (ref.) | 0.7109 | 1 (ref.) | 0.7288 | 1 (ref.) | 0.7692 | 1 (ref.) | 0.817 |
|  | Yes | 8220 | 2 | 60413.61 | 0.033105 | 1.337 (0.288, 6.195) |  | 1.316 (0.279, 6.212) |  | 1.262 (0.266, 5.986) |  | 0.826 (0.163, 4.185) |  |
| Nerve and root (G50-59) | No | 41100 | 1 | 354903.85 | 0.002818 | 1 (ref.) | - | 1 (ref.) | - | 1 (ref.) | - | 1 (ref.) | - |
|  | Yes | 8220 | 0 | 60413.61 | 0 | - |  | - |  | - |  | - |  |
| Peripheral (G60-64))) | No | 41100 | 2 | 354903.85 | 0.005635 | 1 (ref.) | - | 1 (ref.) | - | 1 (ref.) | - | 1 (ref.) | - |
|  | Yes | 8220 | 0 | 60413.61 | 0 | - |  | - |  | - |  | - |  |
| Muscle (G70-73) | No | 41100 | 0 | 354903.85 | 0 | 1 (ref.) | - | 1 (ref.) | - | 1 (ref.) | - | 1 (ref.) | - |
|  | Yes | 8220 | 1 | 60413.61 | 0.016553 | - |  | - |  | - |  | - |  |
| Cerebral palsy (G80-83) | No | 41100 | 2 | 354903.85 | 0.005635 | 1 (ref.) | - | 1 (ref.) | - | 1 (ref.) | - | 1 (ref.) | - |
|  | Yes | 8220 | 0 | 60413.61 | 0 | - |  | - |  | - |  | - |  |
| Others (G90-99) | No | 41100 | 7 | 354903.85 | 0.01972 | 1 (ref.) | 0.0008 | 1 (ref.) | 0.0006 | 1 (ref.) | 0.0011 | 1 (ref.) | 0.0033 |
|  | Yes | 8220 | 7 | 60413.61 | 0.11587 | 6.072 (2.125, 17.352) |  | 6.657 (2.241, 19.773) |  | 6.148 (2.072, 18.241) |  | 5.602 (1.776, 17.672) |  |
